# Pre- and Post-therapy Assessment of Clinical Outcomes and White Matter Integrity in Autism Spectrum Disorder: Pilot Study

**DOI:** 10.3389/fneur.2019.00877

**Published:** 2019-08-13

**Authors:** Stephanie Saaybi, Natally AlArab, Salem Hannoun, Maritherese Saade, Rayyan Tutunji, Carine Zeeni, Rolla Shbarou, Roula Hourani, Rose-Mary Boustany

**Affiliations:** ^1^Division of Pediatric Neurology, Departments of Pediatrics and Adolescent Medicine/Biochemistry and Molecular Genetics, American University of Beirut Medical Center, Beirut, Lebanon; ^2^Department of Pediatrics and Adolescent Medicine, MedStar Georgetown University Hospital, Washington, DC, United States; ^3^Division of Neuroradiology, Department of Diagnostic Radiology, American University of Beirut Medical Center, Beirut, Lebanon; ^4^Faculty of Medicine, Nehme and Therese Tohme Multiple Sclerosis Center, American University of Beirut Medical Center, Beirut, Lebanon; ^5^Faculty of Medicine, Abu-Haidar Neuroscience Institute, American University of Beirut Medical Center, Beirut, Lebanon; ^6^AUBMC Special Kids Clinic (ASKC), American University of Beirut Medical Center, Beirut, Lebanon; ^7^Cognition and Behaviour, Department of Cognitive Neuroscience, Donders Institute for Brain, Radboud University Medical Centre, Nijmegen, Netherlands; ^8^Division of Neuro-Anesthesia, Department of Anesthesia, American University of Beirut Medical Center, Beirut, Lebanon

**Keywords:** diffusion tensor imaging (DTI), autism spectrum disorder (ASD), applied behavior analysis (ABA) therapy, ASD interventional therapies, VB-MAPP assessment

## Abstract

**Objective:** This pilot study aims to identify white matter (WM) tract abnormalities in Autism Spectrum Disorders (ASD) toddlers and pre-schoolers by Diffusion Tensor Imaging (DTI), and to correlate imaging findings with clinical improvement after early interventional and Applied Behavior Analysis (ABA) therapies by Verbal Behavior Milestones Assessment and Placement Program (VB-MAPP).

**Methods:** DTI scans were performed on 17 ASD toddlers/pre-schoolers and seven age-matched controls. Nine ASD patients had follow-up MRI 12 months following early intervention and ABA therapy. VB-MAPP was assessed and compared at diagnosis, 6 and 12 months after therapies. Tract-Based Spatial Statistics (TBSS) was used to measure fractional anisotropy (FA), mean diffusivity (MD), axial diffusivity (AD), and radial (RD) diffusivity.

**Results:** VB-MAPP scores improved at 6 and 12 months after early intervention and ABA therapy compared to scores at baseline. TBSS analysis showed significant FA decrease and/or RD increase in ASD patients before therapy vs. controls in inferior fronto-occipital fasciculi, uncinate fasciculi, left superior fronto-occipital fasciculus, forceps minor, left superior fronto-occipital fasciculus, right superior longitudinal fasciculus, corona radiate bilaterally, and left external capsule. A significantly FA increase in 21 tracts and ROIs is reported in post- vs. pre-therapy DTI analysis.

**Conclusion:** DTI findings highlighted ASD patient WM abnormalities at diagnosis and confirmed the benefits of 12 months of early intervention and ABA therapy on clinical and neuro imaging outcomes.

## Introduction

Autism spectrum disorders (ASD) are neurodevelopmental disorders characterized by repetitive and restrictive behaviors, and language and socialization deficits in early childhood (1). Increased parent/physician awareness coupled with improved assessment tools, lead to better diagnosis, and higher prevalence [1/40 children in the United States (2) and 1/66 children in Lebanon] (3). Parents typically have concerns in the first year of life, but diagnosis of ASD is made between the ages of 18–24 months.

Genetic and environmental factors, including prenatal, and perinatal complications are important contributors to ASD risk (4). An abnormal developmental trajectory of the brain (5), early brain development and changes in gray and WM structures and connectivity are also associated with emergence of symptoms (6). WM tracts structural integrity and change in myelination contribute to the pathogenesis of ASD shown in several age-groups (7, 8). In addition to lack of knowledge regarding myelination in toddlers with ASD, contradictions abound in the literature as to which specific areas are implicated.

New tools for early diagnosis and prediction in high-risk populations, based on MRI data have been identified (8). DTI offers insight into the micro-structural organization and integrity of WM (9). DTI studies performed on infants and young children report increased FA in frontal tracts, left uncinate fasciculus, inferior longitudinal fasciculus and corpus callosum at 6 months of age (7). Longitudinal studies by Wolff et al. and Solso et al. in infants and toddlers state that ASD has a dynamic neural pathology (7, 10). Abnormal fractional anisotropy (FA) in ASD has been reported in pathways affecting socio-emotional processing in the limbic system, language areas, temporal lobes, and higher order functions involving the frontal cortex (11). These regions identify childhood ASD as a disorder of fronto-temporal-limbic circuitry (12). Additionally, decreased FA, increased mean and radial diffusivities (MD, RD) in the corpus callosum (CC) in young ASD patients (10, 13), are probably associated with lower intelligence quotients and slower processing rates.

The standard of care approach for toddlers and pre-schoolers with ASD currently includes integration in nurseries as well as a customized curriculum involving regular sessions of psychomotor, speech, occupational and Applied Behavior Analysis therapies. ABA is based on the science of learning and behavior (14). In general, the objectives of ABA in the treatment of children with autism are to increase desirable behaviors, decrease undesirable behaviors, and increase skill acquisition in every area of development (language and communication, social, academic, daily living skills, motor skills, etc.). Since the 1960s, therapists have been applying behavior analysis to help children with autism and related developmental disorders. Several papers provide evidence of the effectiveness of ABA treatment for children with autism (15). Dawson et al. (16) conducted the first trial to demonstrate the effect of early behavioral intervention on brain activity. The results of EEG in ASD patients between 18 and 30 months compared to age-matched controls suggest that early interventional therapies are associated with normalized brain activity patterns related to social attention and engagement, and that these normalized brain activity patterns are correlated with improvements in social behavior.

The clinical effect of these therapies in ASD patients is documented in the literature (17). However, its biologic impact on WM microstructure has not been addressed. The aims of this study are to characterize the impact of early intervention/behavioral therapies on WM integrity in specific brain regions in autistic toddlers and pre-schoolers. In this pilot study, ASD patients underwent MRI scans and behavioral assessment using The Verbal Behavior Milestones Assessment and Placement Program (VB-MAPP), a comprehensive evaluation which is designed to identify the existing language and related developmental skills for children with autism or other developmental disabilities, before and after therapy, to explore the positive impact of therapies on imaging and clinical outcomes.

## Methods

### Subjects

Recruitment of participants took place at the American university of Beirut Medical Center (AUBMC), between 2014 and 2018. Participants were toddlers and pre-schoolers between the age of 18 months and 4 years.

Children with contraindications to anesthesia or history of neurological disorders, infection, ischemia, or genetic disorders were excluded. Diagnosis of patients was based on the Diagnostic and Statistical Manual of Mental Disorders (DSM-V) criteria by two experienced pediatric neurologists at AUBMC. The Autism Diagnostic Observation Schedule (ADOS) conducted by behavioral and speech therapists helped confirm the diagnosis. The group of ASD children was heterogeneous with inclusion of all severity groups (mild, moderate, severe). Asperger and pervasive developmental disorder-not otherwise specified (PDD-NOS) cases were not included.

Additionally, normal developing age-matched controls were recruited at the radiology department of our institution. Children presenting with any symptom requiring imaging by MRI including headaches, suspected new onset seizures, trauma, ophthalmic problems, or orbital disease, hearing or inner ear problems, neck masses, cervical neck pain, or malformation were included. All controls were subjected to initial clinical evaluation by their treating physician and categorized as neuro-typical children prior to being approached and enrolled in this study.

Subjects with prematurity (<36 weeks gestation), any medical condition affecting brain development, any motor deficits, sensory impairment, psychiatric disorder as well as adopted children with no available prenatal and perinatal history were excluded. In addition, the presence of any abnormal findings on brain MRI such as tumors or congenital malformations or a leukodystrophy, resulted in withdrawing subjects from the study.

### Behavioral Assessment and Early Intervention Therapies

In ASD group 1 (G1), once the diagnosis of ASD was established, patients underwent a Verbal Behavior Assessment and Placement Program (VB-MAPP) assessment. This is a criterion-referenced evaluation battery that guides therapy and serves to follow-up progression of skills in children with ASD and language delays (18) VB-MAPP assesses developmental communicative milestones. Patients underwent VB-MAPP evaluations at initial diagnosis, 6 and 12 months after initiation of ABA behavior analysis and early intervention therapies.

Treatment consisted of attending nursery school 5 days a week, 6–10 h of ABA sessions in homes and/or nurseries per week, in addition to 1 h each of speech, occupational and psychomotor therapy. Patients underwent two MRI examinations, one before beginning early intervention and the second repeated 12 months after therapy. ASD patients re-scanned 12 months later were referred to as group 2 (G2).

### Image Acquisition

MRI image acquisition was performed on a 3 Tesla Phillips Ingenia MR system (Philips Healthcare, Best, The Netherlands). All subjects underwent sedation for the scan. Oral chloral hydrate was used in r 4/7 children in the control group and 4 children in ASD group at baseline, or deep sedation administered by an anesthesiologist for remaining controls and ASD patients before and after therapies). The conventional MR imaging protocol consisted in the acquisition of 3D sagittal T1 turbo field echo sequence without gadolinium injection with echo time/repetition time (TE/TR) = 3.8/8.3 ms, flip angle = 8°, field of view (FOV) of 240 × 240 mm, matrix of 240 × 222 and a spatial resolution of 1 × 0.94 × 0.94 mm^3^. A 3D (FLAIR) fluid attenuated inversion recovery image with echo time/repetition time/inversion time = 347/4,800/1,660 ms, FOV = 220 × 220 mm, slice thickness = 1 mm (with no gap), and a matrix of 224 × 224.

DTI data were acquired using a multi-slice echo-planar spin-echo sequence (TR/TE = 11279.5/106 ms). Seventy-five contiguous, 2 mm thick, axial slices were acquired covering the whole brain. The protocol comprised 32 non-collinear diffusion gradient directions with b values of 0 and 1,000 s/mm^2^. A 1.75 × 1.75 × 2 mm^3^ resolution was achieved with a matrix size of 112 × 112 over a FOV of 128 × 128 mm.

DTI data was visually inspected (by SH and RH) in order to detect artifacts arising from subject motion or scanner malfunction and to confirm the lack of visually detectable abnormalities.

### Data Processing

The FMRIB Software Library (FSL) (19) was used for the analysis of DTI data. An Eddy current correction followed by a non-brain voxels extraction was applied. Diffusivity maps (FA, axial diffusivity (AD), RD, and MD) were generated and checked for presence of significant residual motion or other artifacts.

FA maps were then grouped for the tract-based spatial statistics (TBSS) analysis (20). All subject data were initially non-linearly aligned to one of the subjects' space. Since the study cohort consists of children, the common template used in FSL was thereby not adapted. To this end, all subjects were registered to one another in order to find the most “typical” subject to use as a target image to align all others. All registered FA maps were averaged to obtain a mean FA image that was later used to generate a mean skeleton of the major WM tracts on which all aligned subjects FA data were projected. The resulting data were then fed into a voxel-wise statistical analysis (described below), performed to identify significant differences. For better assessment, TBSS was also applied to the other diffusivity maps (AD, RD, and MD). DTI metrics differences were evaluated between the patient group before treatment (G1) and the control group and between patients themselves before and after treatment (using age and time interval between both scans as well as sex of the patients as covariates).

Voxel-wise analysis was based on a non-parametric approach using the permutation test theory with a standard generalized linear model design matrix. The permutation testing was performed using the Randomize module of FSL (21). The threshold-free cluster enhancement (TFCE) option of Randomize was applied on the resulting statistical maps that were corrected for multiple comparisons. The anatomical location of significant clusters was identified based on WM atlases (JHU ICBM-DTI81 White Mater Labels and JHU White-Matter Tractography Atlas) in FSL. In order to confirm the voxel-wise analysis results, quantitative values of diffusion metrics were extracted from 48 regions of interest (ROI) and 21 fiber-bundles of the JHU atlases, by multiplying atlas labels with the TBSS skeleton obtained from all 24 subjects (22). ROI data extraction was solely performed on the significant masks obtained from the TBSS analysis. To this end, a 5% value of significant voxels per total number of voxels per ROI was set as a threshold to consider the ROI significant.

### Ethical Approval

This study protocol was approved by the Institutional Review Board of AUBMC. Written informed consent was obtained for all participants from parents and/or legal guardians in accordance with the Declaration of Helsinki.

### Statistical Analysis

The Statistical Package for Social Sciences (SPSS), version 24.0 was used for data cleaning, management, and analyses. Descriptive statistics including mean, median, range, standard deviation, and frequencies with percentages were calculated. Statistical analysis was performed using the Mann Whitney for continuous variables and Fisher exact test for categorical variables. The Wilcoxon Signed rank test was used to compare the VB-MAPP scores at diagnosis, after 6 and 12 months post-therapy. Results are presented with a 95% Confidence interval (CI). Significant *P* < 0.05.

## Results

### Demographics

Newly diagnosed ASD patients (G1, *n* = 17) had a median age of 2.9 years (1.9–4.7). Nine patients repeated the MRI examination 12 months after initiating therapy (G2) and had a median age of 4.2 years (2.1–6). Seven control subjects were included in the study with a median age of 3.3 years (1.6–4.7). No significant differences in age between males and females were observed between G1 ASD patients and normal controls (*p* = 0.261) as well as between G2 and G1 ASD patients (*p* = 1) (Table 1).

**Table 1:** Demographics of normal pediatric controls, 17 patients with ASD at diagnosis (G1), and nine patients with ASD after 12-months of therapy (G2).

	**Normal controls** **(NC) *N* = 7**	**Autism patients at diagnosis** **(G 1) *N* = 17**	**Autism patients** **after 12 months of therapies *N* = 9 (G2)**	**NC & G1**
Age (years)	**Median (min-max)**	**Median (min-max)**	**Median (min-max)**	***P*****-value**
	3.3 (1.6–4.7)	2.9 (1.9–4.7)	4.2 (2.1–6.0)	0.227^a^
Sex	**Frequency (%)**	**Frequency (%)**	**Frequency (%)**	***P*****-value**
Males	4 (57.1)	12 (70.6)	5 (55)	0.647^b^
Females	3 (42.9)	5 (29.4)	4 (45)	

a *Mann-Whitney test*;

b *Fisher's exact test*.

### VB-MAPP Scores at Diagnosis, Six and Twelve Months Following Initiation of Therapies in the ASD Group

**Figure 1:**
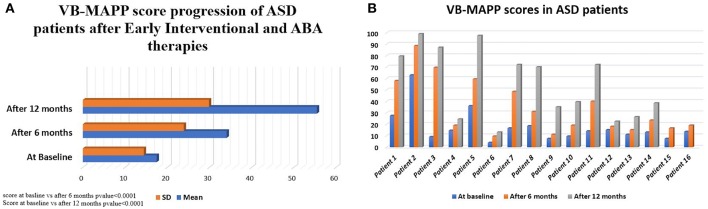
**(A)** VB-MAPP median scores of 16 ASD patients at diagnosis, 14 ASD patients after 6 and 12 months of interventional therapies. The score increased from a median of 13.75 [4-63.59] at diagnosis to reach 21.25 [10-88.78] and 70 [13-99.86] at 6 and 12 months, respectively. Wilcoxon Signed Ranks test was done to detect differences between the 3 scores at diagnosis and at 6 months (*p* < 0.0001), score at 6 months and 12 months (*p* < 0.0001). **(B)** Progression of VB-MAPP scores and improvement of values at diagnosis and over 12 months of therapy in ASD patients (14/16 cases with assessments at diagnosis, 6 and 12 months after therapy, 2/16 cases with first and second assessment at diagnosis and at 6 months).

VB-MAPP scores were closely monitored in the ASD patients. Sixteen of the seventeen enrolled ASD patients underwent VB-MAPP testing with only one patient refusing the assessment. VB-MAPP scores at the time of initial ASD diagnosis had a median score of 13.75 (IQ = 4–63.59) and a mean of 17.5 ± 14.6. All 16 patients completed their 6-months VB-MAPP score assessment with a median of 21.25 (IQ = 10–88.78) and mean of 34.1 ± 24.9. Two patients have not yet completed their 12-months assessment. The median VB-MAPP scores at 12 months after therapies was 70 (IQ = 13–99.86) with a mean of 55.47 ± 29.9. This confirms significant improvement in VB-MAPP scores at 6 months of early interventional and ABA therapies compared to baseline scores (*p* < 0.0001) and at 12 months of therapies compared to initial scores at diagnosis (*p* < 0.0001) (Figure 1A). This clinical improvement was documented when comparing VB-MAPP scores of each patient individually (Figure 1B).

### TBSS Analyses

#### Comparison Between ASD Groups (G1/G2) and Neurotypical Controls

TBSS analysis showed significant differences in FA and RD when comparing ASD patients at baseline (G1) to the control group. FA was significantly decreased in ASD patients compared to controls in left and right inferior fronto-occipital fasciculi, left and right uncinate fasciculi, left superior fronto-occipital fasciculus, left anterior corona radiata, and left external capsule. RD was significantly increased in ASD patients compared to controls in the forceps minor, left superior fronto-occipital fasciculus, right inferior fronto-occipital fasciculus, left and right uncinate fasciculi, right superior longitudinal fasciculus (temporal part), right superior longitudinal fasciculus, left and right anterior corona radiata, and left external capsule. No significant differences were observed in AD and MD between both groups (Table 2).

**Table 2:** White matter tracts with lower values (>5%, *p* < 0.05) in fractional anisotropy (FA) and/or higher values in radial diffusivity (RD) in ASD patients at diagnosis (G1) compared to controls by TBSS.

**White matter tracts**	**Fractional anisotropy**			**Radial diffusivity**		
	**% of significant FA regional differences**	**FA controls** **(*N* = 7)** **Mean ± SD**	**FA patients at diagnosis (*N* = 17)** **Mean ± SD**	**% of significant RD regional differences**	**RD controls** **(*N* = 7)** **Mean ± SD**	**RD patients at diagnosis (*N* = 17)** **Mean ± SD**
**JHU ATLAS**						
LUF	10.41	0.406 ± 0.025	0.373 ± 0.027	8.83	0.649 ± 0.030	0.687 ± 0.033
RUF	7.91	0.439 ± 0.026	0.392 ± 0.020	11.61	0.630 ± 0.031	0.678 ± 0.028
LIFOF	5.94	0.421 ± 0.017	0.391 ± 0.028	5.49	0.635 ± 0.022	0.675 ± 0.047
Fminor	4.85	0.538 ± 0.031	0.506 ± 0.036	5.57	0.535 ± 0.039	0.585 ± 0.054
RSLF^*^	0	0.534 ± 0.027	0.491 ± 0.039	13.42	0.548 ± 0.030	0.613 ± 0.049
**ICBM ATLAS**						
LSFOF^*^	13.02	0.455 ± 0.049	0.419 ± 0.035	0	0.548 ± 0.045	0.583 ± 0.043
RIFOF	11.84	0.458 ± 0.019	0.441 ± 0.036	13.68	0.639 ± 0.022	0.676 ± 0.052
LACR	10.06	0.410 ± 0.024	0.375 ± 0.034	9.24	0.634 ± 0.032	0.686 ± 0.055
LEC	5.76	0.376 ± 0.021	0.354 ± 0.025	3.49	0.658 ± 0.023	0.683 ± 0.032
RACR	4.85	0.402 ± 0.023	0.375 ± 0.024	7.74	0.639 ± 0.032	0.681 ± 0.045
RSLF	1.32	0.429 ± 0.018	0.399 ± 0.028	6.10	0.611 ± 0.020	0.651 ± 0.043

LUF, Left uncinate fasciculus; RUF, Right uncinate fasciculus; LIFOF, Left inferior fronto-occipital fasciculus; RSLF, Right superior longitudinal fasciculus; Fminor, Forceps minor; LSFOF^*^, Left superior fronto-occipital fasciculus (may be part of anterior internal capsule); RIFOF, Right inferior fronto-occipital fasciculus; LACR, Left anterior corona radiata; LEC, Left external Capsule; RACR, Right anterior corona cadiata; RSLF^*^, Right superior longitudinal fasciculus.

#### Paired Comparison Between ASD Subgroups at Diagnosis (G1) and 12 Months Post-therapies (G2)

TBSS analysis was also performed on the ASD group (*n* = 9) after completing a 12-months treatment period and had underwent pre- and post-treatment MRIs. Age and time in between the two scans were used as a covariate. A significant increase in FA was observed in the post-treatment ASD group compared to baseline values in the following areas: left and right superior longitudinal fasciculi/left and right inferior fronto-occipital fasciculi/left and right corticospinal tracts/left and right anterior thalamic radiations/forceps major/forceps minor/left external capsule/left and right anterior and posterior corona radiate/right sagittal stratum/left and right posterior and anterior limbs of internal capsule/left cerebral peduncle/right fornix cres/stria terminalis/splenium/genu, and body of the corpus callosum (CC) (Table 3). No significant changes were observed for AD, RD, and MD.

**Table 3:** White matter tracts with higher values (>5%, *p* < 0.05) in fractional anisotropy (FA) in ASD patients after 12 months of therapies (G2) compared to patients at diagnosis (G1) by TBSS.

**White matter tracts**	**% of significant FA regional differences post-therapy**	**FA patients at diagnosis (*N* = 9)** **Mean ± SD**	**FA patients after therapies (*N* = 9)** **Mean ± SD**
**JHU ATLAS**			
RIFOF	32.268	0.405 ± 0.033	0.428 ± 0.031
LCST	32.086	0.544 ± 0.026	0.566 ± 0.026
Fminor	31.589	0.493 ± 0.039	0.515 ± 0.039
RCST	30.898	0.561 ± 0.028	0.584 ± 0.028
RATR	26.682	0.366 ± 0.020	0.381 ± 0.024
RSLF	18.736	0.382 ± 0.024	0.394 ± 0.029
LIFOF	16.225	0.383 ± 0.030	0.408 ± 0.033
Fmajor	14.101	0.416 ± 0.044	0.449 ± 0.043
LATR	9.172	0.371 ± 0.023	0.391 ± 0.028
LSLF	7.330	0.414 ± 0.068	0.438 ± 0.069
**ICBM ATLAS**			
BCC	46.51	0.592 ± 0.040	0.612 ± 0.032
LRLIC	43.21	0.506 ± 0.033	0.531 ± 0.036
RRLIC	42.23	0.483 ± 0.040	0.495 ± 0.041
RPCR	39.79	0.412 ± 0.026	0.424 ± 0.029
LACR	38.42	0.365 ± 0.039	0.393 ± 0.042
RPTR^*^	36.94	0.489 ± 0.046	0.522 ± 0.041
RACR	34.09	0.368 ± 0.029	0.392 ± 0.032
GCC	30.66	0.638 ± 0.040	0.657 ± 0.037
LPCR	26.32	0.421 ± 0.037	0.442 ± 0.034
RALIC	21.69	0.476 ± 0.019	0.497 ± 0.024
LEC	21.25	0.346 ± 0.022	0.370 ± 0.018
RSLF	15.26	0.394 ± 0.026	0.405 ± 0.028
LPLIC	14.37	0.613 ± 0.027	0.636 ± 0.026
SCC	13.31	0.679 ± 0.015	0.694 ± 0.020
Fx (cres)/ RST	10.56	0.432 ± 0.037	0.441 ± 0.038
LCP	9.90	0.561 ± 0.019	0.591 ± 0.022
RPLIC	9.77	0.626 ± 0.027	0.642 ± 0.020
RSS^*^	6.10	0.432 ± 0.038	0.458 ± 0.0.40
LSLF	5.77	0.396 ± 0.021	0.041 ± 0.023

LSLF, Left Superior longitudinal fasciculus; RSLF, Right Superior longitudinal fasciculus; Fminor, Forceps minor; LIFOF, Left Inferior fronto-occipital fasciculus; Fmajor, Forceps major; RIFOF, Right Inferior fronto-occipital fasciculus; LCST, Left corticospinal tract; RCST, Right corticospinal tract; LATR, left anterior thalamic radiation; BCC, Body of corpus callosum; RATR, Right anterior thalamic radiation; LEC, Left external capsule; Fx, Fornix (cres); LCP, Left Cerebral peduncle, LACR, Left anterior corona radiata; RACR, Right anterior corona radiata; RSS^*^, Right sagittal stratum (inferior longitudinal fasciculus and inferior fronto-occipital fasciculus); RPLIC, Right Posterior limb internal capsule; RST, Right stria terminalis; GCC, Genu of corpus callosum; SCC, Splenium of corpus callosum; RPCR, Right posterior corona radiata; RPTR^*^, Right posterior thalamic radiation (plus optic radiation); LPCR, Left posterior corona radiata; RRIC, Right retro-lenticular internal capsule; LRLIC, Left retro-lenticular internal capsule, RALIC, Right anterior limb of internal capsule; LPLIC, Left posterior limb of internal capsule.

## Discussion

**Figure 2:**
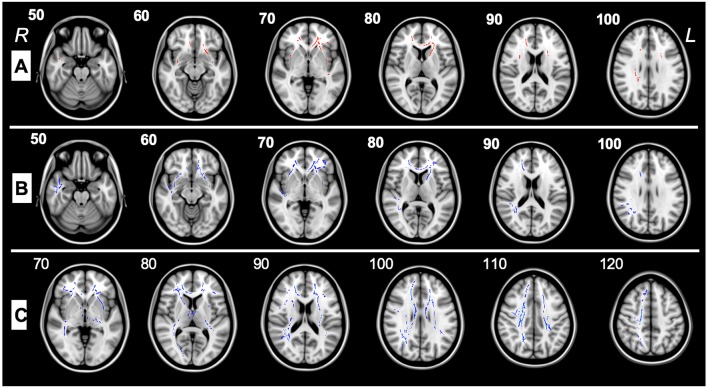
**(A)** TBSS analysis of major WM tracts illustrating significant regional fractional anisotropy (FA) decreases (>5%, *p* < 0.05) shown in red between pre-therapy ASD patients(G1) and control subjects in the left (L) and right (R) uncinate fasciculus, left and right inferior fronto-occipital fasciculus, right superior longitudinal fasciculus, forceps minor, left superior fronto-occipital fasciculus left and right anterior corona radiata, left external capsule, and right superior longitudinal fasciculus. **(B)** TBSS analysis of major WM tracts, illustrating regional radial diffusivity (RD) increases (>5%, *p* < 0.05) shown in blue between G1 and control patients in forceps minor, left superior fronto-occipital fasciculus, right inferior fronto-occipital fasciculus, left and right uncinate fasciculi, right superior longitudinal fasciculus (temporal part), right superior longitudinal fasciculus, left and right anterior corona radiata, and left external capsule. **(C)** TBSS analysis of the major WM tracts, illustrating regional fractional anisotropy (FA) increases (>5%, *p* < 0.05) shown in blue between G1 and G2 patients in left and right superior longitudinal fasciculi, left and right inferior fronto-occipital fasciculi, left and right corticospinal tracts, left and right anterior thalamic radiations, forceps major, forceps minor, left external capsule, left and right anterior and posterior corona radiata, right sagittal stratum, left and right posterior and anterior limbs of internal capsule, left cerebral peduncle, right fornix cres, stria terminalis, splenium, genu, and body of the corpus callosum.

This study examines WM differences in ASD using DTI, which enables quantitation of WM maturation in toddlers and pre-schoolers and depicts axonal abnormalities providing symptom interpretation in ASD. DTI/TBSS analysis performed between the ASD group pre-therapy and control subjects highlighted a decreased FA in the frontal regions, the anterior corona radiata, and the external capsule (Figure 2A). Increased RD was also reported in multiple regions involving several frontal pathways, the inferior corona radiata, the left external capsule and the forceps minor (Figure 2B). FA was found to be significantly higher in ASD patients after therapy as compared to themselves before therapy in 21 tracts and ROIs (Table 3).

The significant difference between the ASD patients at baseline and neurotypical controls may explain the delay in neural connectivity between various brain regions, which in turn contributes to emotional lability, restricted social communication, language delay, and other symptoms observed in ASD. Decreased FA could be the outcome of several mechanisms including decreased myelination/axonal density and abnormal axonal organization (23), whereas increased RD, may reflect differences in myelination, axonal diameter or packing density and/or glial densities (24). These results are in line with previous reports of reduced FA in the WM of ASD patients indicating lower coherence of directionality in several regions such as the prefrontal cortices, temporal-parietal junctions, fronto-temporal/fronto-parietal tracts and the CC (23, 25). A study evaluating development of WM tracts in infants at high risk for ASD shows increased FA in several tracts from 6 till 24 months of age in participants without ASD symptoms, and higher values than in ASD-confirmed patients (7). Other studies, however, demonstrate increased FA and decreased RD in children with ASD in multiple regions including frontal pathways, CC, posterior limb of internal capsule, external capsule, and the superior longitudinal fasciculus (10, 26). Symptoms of ASD imply over-connectivity and increase in myelination in tracts connecting different brain regions, thus, altering the coherence of axon orientation in those children. At present, there is no definite pathophysiological process that explains the neurodevelopmental delay in ASD toddlers.

This study goes beyond comparing controls and ASD patients at diagnosis. It employed VB-MAPP and DTI over time, to measure the effectiveness of interventions on developmental outcomes and white matter integrity in ASD patients. Nine of seventeen patients (G2) underwent re-scanning after 12 months of ABA and other interventional therapies. TBSS analysis was performed on G2 scans (from post-therapy patients) and compared to pre-therapy scans.

Pre- and post-therapy analysis established improvement of WM integrity through the documented increase in FA (Table 3). WM pathways rapidly develop during the first years of life (27, 28). In the first 5 years of life, a gradual increase in FA and decrease in MD is observed. After this age, FA changes are less conspicuous and are attributed to completion of mechanisms of myelination and alterations in intra-axonal microstructure, caliber, and orientation (28–31). FA values in normal developing infants show rapid change from 3–6 months of age followed by slower change until 24 months. At 48 months of age, FA color maps seems almost identical to the ones observed in adults (29). This underscores the importance of timely imaging and clinical diagnosis of ASD patients with early implementation of interventional therapies. It is noteworthy to mention that patients evaluated at G1 and G2 had a mean age of 2.9 and 4.2 years, respectively. Thus, minor differences are to be expected because of the slow rate of neurodevelopmental maturation of neural tracts between 2–5 years of age. So far, a clear increase in FA in multiple tracts post-therapy (Figure 2C) was observed. We hypothesize that the early implementation of ABA, occupational, psychomotor and speech therapies aid in brain development and clinical outcome of G2 patients. This is demonstrable by the significant increase in VB-MAPP scores at 6 months and 12 months post-therapies (Figure 1A).

Based on ASD symptoms at diagnosis including delayed or absent speech, poor social communication skills, obsessive behavior, restricted interests, hypersensitivity to sound, light, and other sensory stimulation, TBSS ROI and fiber analysis results of decreased FA and increased RD in the inferior fronto-occipital fasciculus (IFOF) and uncinate fasciculus are expected (Table 2). The IFOF connects ventral occipital lobe and orbitofrontal cortex, and participates in reading, attention and visual processing (32), whereas the uncinate fasciculus is part of the limbic system involved in emotional processing, memory and language functions (32). The FA increase after 12 months of therapy suggests improved myelination and development of the IFOF (Table 3).

In contrast, FA increase and RD decrease in the corona radiata when comparing G1 and G2 patients. The corona radiata regroups fibers transiting from thalamus to cerebral cortex and from fronto-parietal cortex to the subcortical nuclei and then to spinal cord and these form the backbone of perceptual, motor and higher cognitive functions (32). These results underscore the effectiveness of ABA/other interventional therapies and positive impact on motor and perceptual functions controlled by the above-mentioned tracts and regions.

The superior longitudinal fasciculus (SLF) and its connections with cortical regions are involved in language comprehension (Wernicke's area) and production (Broca's area). Studies addressed this tract in children with language delay, a prime symptom in ASD (33). RD was higher in ASD in the right SLF. After 12 months of speech therapy, FA was higher in the SLF in patients compared to themselves before therapy, similarly echoed by improvement in VB-MAPP scores.

The corpus callossum (CC) enables communication between both hemispheres. The CC has been linked to motor skills, working memory, complex information, and speed processing (5). FA decrease in the CC of ASD patients with increase in FA after therapy, may indicate WM deficits causing impaired cortical connectivity between the two hemispheres and less axonal myelination at diagnosis with improvement after ABA and other therapies. DTI revealed rapid and highly significant changes in normal brain maturation with a significant increase in FA in the genu of the CC from 0 to 2 years vs. 2–5 years (31). Whereas, FA values of the splenium of the CC show no significant changes after 2 years of age (31). Knowing that patients with G2 scans have a median age of 4.2 years, reported DTI results and patient clinical improvement confirms the positive impact of interventional therapies on WM tract integrity and clinical outcomes. The abnormalities in WM structure provide a neural basis for disrupted systems-level connectivity in ASD (5, 34).

**Figure 3:**
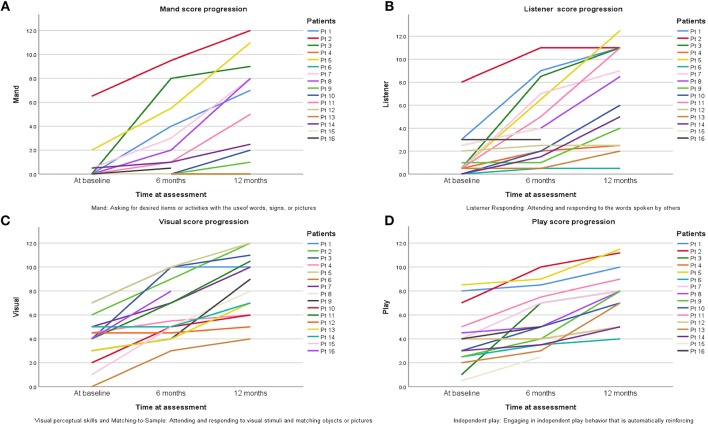
Progress of scores in four VB-MAPP elements assessing the toddlers/pre-schooler ASD milestones at the time of diagnosis, after 6 and 12 months of early interventional treatment. **(A)** Mand reflects if the child has absent, weak, or impaired mand repertoire, in other words “Does the child use words, signs, or pictures to ask for desired items or activities?” **(B)** Listener reflects if the child has absent, weak, or impaired listener repertoires; “Does the child attend to and respond to the words spoken by others?” **(C)** Visual section shows if there is any visual or impaired perceptual skills and matching-to-sample; “Does the child attend to and respond to visual stimuli and match objects or pictures?” **(D)** Play component shows the state of the social and behavioral skills in playing; “Does the child attend to others and attempt to socially engage others?”.

One of the components of the VB-MAPP is the Milestones Assessment, which provides a representative sample of a child's existing verbal and related skills. The assessment includes 170 measurable learning and language milestones that are sequenced and balanced across three developmental levels. Level 1, level 2, and level 3 each h consisting of nine developmental communicative milestones are designed to approximately correspond with the learning and language skills demonstrated by children 0–18 months old, 18–30 months old, and children 30–48 months of age, respectively (18). In this study 150 milestones were measured in total. Two elements of the VB-MAPP measuring a maximum of 20 points (mainly “Echoic” and “Group”) could not be measured. Patients at baseline with a mean age of 2.7 years scored an average of 17.5 ± 14.5 points in their assessment, which corresponds to presence of neuro-developmental delay (reference score = 90 points) thus explaining DTI analysis results in G1 scans compared to controls. After 6 months of ABA and interventional therapies, ASD patients scored an average of 34.1 ± 23.9 points. Although far from the reference score (150 points), progress was documented. Twelve months after therapy, patients scored an average of 55.5 ± 30 points on the VB-MAPP which corresponds to ~37% of the maximum score obtainable at this age. It is worth noting that a huge difference in progress between one ASD child and another will be encountered due to the fact that environmental, genetic, and IQ factors can affect their advancement. The significant progress of VB-MAPP scores in each element of the assessment per child was obvious. The improvement of four important elements of the test are pictured in Figure 3. The progress in VB-MAPP scores was of great value to emphasize the role of early interventional therapies and ABA on clinical improvement in different fields and the development of WM integrity in autistic children in addition to normal physiological development.

## Methodological Strength and Limitations

This is a pilot study and the obtained results are preliminary results. Plans are to continue this longitudinal study and to achieve a higher sample size. The originality of this study relies in being the first of its kind emphasizing the impact of ABA and other interventional therapies on neuroanatomical and clinical improvement of ASD patients using sequential DTI and VB-MAPP assessments over 12-months. Although sample size was small, in light of the young age of participants (1.8–5 years), groups were age and sex matched and clinically similar. In other words, we have carefully considered and ruled out the possibility that group differences were attributable to age and sex. Due to the rare incidence of encountered ASD patients and ethical considerations and the importance of early intervention in ASD, it was not possible to include an additional group of ASD patients not receiving therapies. Future studies will focus on comparing ASD patients after therapy with age-sex matched ASD patients diagnosed at older age and not having received therapies. Due to ethical limitations, scanning control subjects twice with, no valid medical indication was not undertaken. Also, cultural and economic reasons precluded multiple hospital visits for patients and families. Although rare, adverse effects of deep sedation related to ventilation including apnea, desaturation and obstruction, vomiting and aspiration are encountered in medical practice. Thus, to reduce or keep such complications at a minimum excessive sedation was avoided. On the other hand, age-matched controls were used at the outset to control for differences in results not being caused by age and sex differences. Similarly, recruiting older neuro-typical controls can be a future goal. Ideally, a higher number of participants would have allowed assessing effect on individual sub-scales of VB-MAPP in males and females after early interventional and ABA therapies.

## Conclusion

In conclusion, findings highlight WM abnormalities in ASD at diagnosis and confirm the benefits of ABA therapy and early intervention on clinical and neuro imaging outcomes after 12 months. DTI is recommended as a complimentary tool to supplement clinical assessments and aids in monitoring response of ASD to traditional and emerging therapies.

## Data Availability

The datasets for this manuscript are not publicly available because they contain information that could compromise the privacy of research participants. Requests to access datasets should be directed to the corresponding author, rh64@aub.edu.lb.

## Ethics Statement

This study was carried out in accordance with the recommendations and approved by the local ethics committee and was approved by the Institutional Review Board. Written informed consent was obtained for all participants from parents and/or legal in accordance with the Declaration of Helsinki.

## Author Contributions

R-MB, RH, CZ, and RS contributed to the conception and design of the study. SH, SS, RT, MS, and NA contributed to the acquisition and analysis of data. NA, SH, SS, R-MB, and RH contributed to drafting and editing the manuscript text. All authors agreed on the final version of the manuscript.

### Conflict of Interest Statement

The authors declare that the research was conducted in the absence of any commercial or financial relationships that could be construed as a potential conflict of interest.

## Supplementary Material

As mentioned before, one of our limitations was the lack of follow-up for control subjects. Thus, comparing controls at baseline to G2 (Supplementary Data), although controlling for age and sex, is considered flawed. Indeed, in our design, the follow-up exam is the result of three different factors: time (baseline vs. follow-up), group (ASD vs. controls), and intervention (ABA vs. no intervention). The lack of controls follow-up resulted in the loss of the time factor which is particularly important when analyzing the development of the brain in toddlers growing into preschoolers. The results obtained may therefore not be adopted. Nonetheless, the reported DTI results in supplementary data support the effect of ABA and early interventional therapy and are concordant with ASD patients' significant clinical improvement as measured by the VBMAP scores.

Figure S1 TBSS analysis of the major WM tracts, illustrating regional axial diffusivity (AD) decreases (>5%, *p* < 0.05) shown in red between post-therapy ASD patients (G2) and control subjects in left (L) superior longitudinal fasciculus, right (R) superior corona radiata, right retro-lenticular part of internal capsule and the body of the corpus callosum.

## References

[1] Association AP Diagnostic and Statistical Manual of Mental Disorders (DSM-5®). American Psychiatric Pub (2013). 10.1590/s2317-17822013000200017 24413388

[2] Kogan MD Vladutiu CJ Schieve LA Ghandour RM Blumberg SJ Zablotsky B. The prevalence of parent-reported autism spectrum disorder among US children. Pediatrics. (2018) 142:e20174161. 10.1542/peds.2017-4161 30478241 PMC6317762

[3] Chaaya M Saab D Maalouf FT Boustany RM. Prevalence of autism spectrum disorder in nurseries in lebanon: a cross sectional study. J Autism Dev Dis. (2016) 46:514–22. 10.1007/s10803-015-2590-7 26362151

[4] Lyall K Schmidt RJ Hertz-Picciotto I. Maternal lifestyle and environmental risk factors for autism spectrum disorders. Int J Epidemiol. (2014) 43:443–64. 10.1093/ije/dyt282 24518932 PMC3997376

[5] Travers BG Adluru N Ennis C Tromp do PM Destiche D Doran S. Diffusion tensor imaging in autism spectrum disorder: a review. Autism Res. (2012) 5:289–313. 10.1002/aur.1243 22786754 PMC3474893

[6] Oldehinkel M Mennes M Marquand A Charman T Tillmann J Ecker C. Altered connectivity between cerebellum, visual, and sensory-motor networks in autism spectrum disorder: results from the EU-AIMS longitudinal european autism project. Biol Psychiatr. (2018) 4:472–83. 10.1016/j.bpsc.2018.11.014 30711508

[7] Wolff JJ Gu H Gerig G Elison JT Styner M Gouttard S. Differences in white matter fiber tract development present from 6 to 24 months in infants with autism. Am J Psychiatry. (2012) 169:589–600. 10.1176/appi.ajp.2011.11091447 22362397 PMC3377782

[8] Wolff JJ Jacob S Elison JT. The journey to autism: insights from neuroimaging studies of infants and toddlers. Dev Psychopathol. (2018) 30:479–95. 10.1017/S0954579417000980 28631578 PMC5834406

[9] Wakana S Jiang H Nagae-Poetscher LM Van Zijl PC Mori S. Fiber tract–based atlas of human white matter anatomy. Radiology. (2004) 230:77–87. 10.1148/radiol.2301021640 14645885

[10] Solso S Xu R Proudfoot J Hagler DJ Jr Campbell K Venkatraman V. Diffusion tensor imaging provides evidence of possible axonal overconnectivity in frontal lobes in autism spectrum disorder toddlers. Biol Psychiatry. (2016) 79:676–84. 10.1016/j.biopsych.2015.06.029 26300272 PMC4699869

[11] What is AutismDSM-5 Diagnostic Criteria. (2018). Available online at: https://www.autismspeaks.org/what-autism/diagnosis/dsm-5-diagnostic-criteria (assessed April, 2018).

[12] Hull JV Dokovna LB Jacokes ZJ Torgerson CM Irimia A Van Horn JD. Resting-state functional connectivity in autism spectrum disorders: a review. Front Psychiatry. (2017) 7:205. 10.3389/fpsyt.2016.00205 28101064 PMC5209637

[13] Ben Horn D Kronfeld-Duenias V Zachor DA Ekstein PM Hendler T Tarrasch R Accelerated maturation of white matter in young children with autism: a high b value DWI study. Neuroimage. (2007) 37:40–7. 10.1016/j.neuroimage.2007.04.060 17566764

[14] Applied, Behavior Analysis (ABA),. Available online at: https://www.autismspeaks.org/applied-behavior-analysis-aba-0 (accessed May, 2019).

[15] Vismara LA Rogers SJ. Behavioral treatments in autism spectrum disorder: what do we know? Ann Rev Clin Psychol. (2010) 6:447–68. 10.1146/annurev.clinpsy.121208.131151 20192785

[16] Dawson G Jones EJ Merkle K Venema K Lowy R Faja S. Early behavioral intervention is associated with normalized brain activity in young children with autism. J Am Acad Child Adolesc Psychiatry. (2012) 51:1150–9. 10.1016/j.jaac.2012.08.018 23101741 PMC3607427

[17] Peters-Scheffer N Didden R Korzilius H Sturmey P A meta-analytic study on the effectiveness of comprehensive ABA-based early intervention programs for children with autism spectrum disorders. Res Autism Spectr Dis. (2011) 5:60–9. 10.1016/j.rasd.2010.03.011

[18] Sundberg ML VB-MAPP Verbal Behavior Milestones Assessment and Placement Program: A Language and Social Skills Assessment Program for Children With Autism or Other Developmental Disabilities: Guide. Mark Sundberg (2008).

[19] Smith SM Jenkinson M Woolrich MW Beckmann CF Behrens TE Johansen-Berg H. Advances in functional and structural MR image analysis and implementation as FSL. Neuroimage. (2004) 23 (Suppl. 1):S208–19. 10.1016/j.neuroimage.2004.07.051 15501092

[20] Smith SM Jenkinson M Johansen-Berg H Rueckert D Nichols TE Mackay CE. Tract-based spatial statistics: voxelwise analysis of multi-subject diffusion data. Neuroimage. (2006) 31:1487–505. 10.1016/j.neuroimage.2006.02.024 16624579

[21] Winkler AM Ridgway GR Webster MA Smith SM Nichols TE. Permutation inference for the general linear model. NeuroImage. (2014) 92:381–97. 10.1016/j.neuroimage.2014.01.060 24530839 PMC4010955

[22] Nusbaum F Hannoun S Kocevar G Stamile C Fourneret P Revol O. Hemispheric differences in white matter microstructure between two profiles of children with high intelligence quotient vs. controls: a tract-based spatial statistics study. Front Neurosci. (2017) 11:173. 10.3389/fnins.2017.00173 28420955 PMC5376583

[23] Sundaram SK Kumar A Makki MI Behen ME Chugani HT Chugani DC. Diffusion tensor imaging of frontal lobe in autism spectrum disorder. Cerebral Cortex. (2008) 18:2659–65. 10.1093/cercor/bhn031 18359780 PMC2567426

[24] Alexander AL Lee JE Lazar M Boudos R DuBray MB Oakes TR. Diffusion tensor imaging of the corpus callosum in Autism. Neuroimage. (2007) 34:61–73. 10.1016/j.neuroimage.2006.08.032 17023185

[25] Jeong J-W Kumar A Sundaram SK Chugani HT Chugani DC. Sharp curvature of frontal lobe white matter pathways in children with autism spectrum disorders: tract-based morphometry analysis. Am J Neuroradiol. (2011) 32:1600–6. 10.3174/ajnr.A2557 21757519 PMC3868442

[26] Weinstein M Ben-Sira L Levy Y Zachor DA Itzhak EB Artzi M. Abnormal white matter integrity in young children with autism. Hum Brain Map. (2011) 32:534–43. 10.1002/hbm.21042 21391246 PMC6870180

[27] Yoshida S Oishi K Faria AV Mori S. Diffusion tensor imaging of normal brain development. Pediatr Radiol. (2013) 43:15–27. 10.1007/s00247-012-2496-x 23288475 PMC3703661

[28] Hasan KM Sankar A Halphen C Kramer LA Brandt ME Juranek J. Development and organization of the human brain tissue compartments across the lifespan using diffusion tensor imaging. NeuroReport. (2007) 18:1735–9. 10.1097/WNR.0b013e3282f0d40c 17921878

[29] Hermoye L Saint-Martin C Cosnard G Lee S-K Kim J Nassogne M-C. Pediatric diffusion tensor imaging: normal database and observation of the white matter maturation in early childhood. Neuroimage. (2006) 29:493–504. 10.1016/j.neuroimage.2005.08.017 16194615

[30] Cascio CJ Gerig G Piven J. Diffusion tensor imaging: application to the study of the developing brain. J Am Acad Child Adolesc Psychiatry. (2007) 46:213–23. 10.1097/01.chi.0000246064.93200.e8 17242625

[31] Ding X-Q Sun Y Braaß H Illies T Zeumer H Lanfermann H. Evidence of rapid ongoing brain development beyond 2 years of age detected by fiber tracking. Am J Neuroradiol. (2008) 29:1261–5. 10.3174/ajnr.A1097 18436615 PMC8119151

[32] Catani M Thiebaut de Schotten M. A diffusion tensor imaging tractography atlas for virtual *in vivo* dissections. Cortex. (2008) 44:1105–32. 10.1016/j.cortex.2008.05.004 18619589

[33] Madhavan KM McQueeny T Howe SR Shear P Szaflarski J. Superior longitudinal fasciculus and language functioning in healthy aging. Brain Res. (2014) 1562:11–22. 10.1016/j.brainres.2014.03.012 24680744 PMC4049076

[34] Just MA Keller TA Malave VL Kana RK Varma S. Autism as a neural systems disorder: a theory of frontal-posterior underconnectivity. Neurosci Biobehav Rev. (2012) 36:1292–313. 10.1016/j.neubiorev.2012.02.007 22353426 PMC3341852

