# Supplementary material for: Pre- and Post-therapy Assessment of Clinical Outcomes and White Matter Integrity in Autism Spectrum Disorder: Pilot Study
Source: Front Neurol. 2019 Aug 13;10:877. doi: 10.3389/fneur.2019.00877 (PMC6701406; doi:10.3389/fneur.2019.00877)
Supplement: Supplementary file 1 [file Data_Sheet_1.docx]

Supplementary data:

When comparing post-therapy ASD patients (G2) to baseline control subjects, TBSS analysis showed significant decrease in AD only in the left superior longitudinal fasciculus (G2=0.208±0.0034, controls=1.159±0.037), right superior corona radiata (G2=1.219±0.049, controls=1.174±0.030), right retro-lenticular part of internal capsule (G2=1.136±0.042, controls=1.318±0.043), and in the body of the corpus callosum (G2=1.596±0.061, controls =1.509±0.061).( supplementary figure). The lack of FA and RD changes implies that ASD patients are similar to neuro-typical controls in WM integrity and connectivity measured by DTI in multiple tracts after twelve months of therapy (supplementary figure 1). Nonetheless, the discrepancies in the above-mentioned tracts could be related to persistent deficits in verbal communication, cognitive function, social emotional reciprocity and poor eye contact (1). This can be further explained by the fact that improved development in WM in ASD patients could be attributed, in part, to therapy, and, to the normal physiological development of brain. In addition to axonal loss, AD reduction is attributable to changes in axonal number and size (2) and changes in tract compactness (3). Changes in AD cannot be used as a predicting marker of ASD WM abnormalities. Early imaging of ASD patients at the onset of symptoms is necessary as opposed to later (4).

1. Applied Behavior Analysis (ABA) [cited 2019]. Available from: <https://www.autismspeaks.org/applied-behavior-analysis-aba-0>.

2. Harsan LA, Poulet P, Guignard B, Steibel J, Parizel N, Loureiro de Sousa P, et al. Brain dysmyelination and recovery assessment by noninvasive in vivo diffusion tensor magnetic resonance imaging. *Journal of Neuroscience Research* (2006) 83(3):392-402. doi: 10.1002/jnr.20742.

3. Schonberger RB, Worden WS, Shahmohammadi K, Menn K, Silverman TJ, Stout RG, et al. Topical non-iontophoretic application of acetylcholine and nitroglycerin via a translucent patch: a new means for assessing microvascular reactivity. *The Yale journal of biology and medicine* (2006) 79(1):1.

4. Naismith R, Xu J, Tutlam N, Trinkaus K, Cross A, Song S-K. Radial diffusivity in remote optic neuritis discriminates visual outcomes. *Neurology* (2010) 74(21):1702-10.
