# Supplementary figures and images for: Pre- and Post-therapy Assessment of Clinical Outcomes and White Matter Integrity in Autism Spectrum Disorder: Pilot Study
Source: Front Neurol. 2019 Aug 13;10:877. doi: 10.3389/fneur.2019.00877 (PMC6701406; doi:10.3389/fneur.2019.00877)

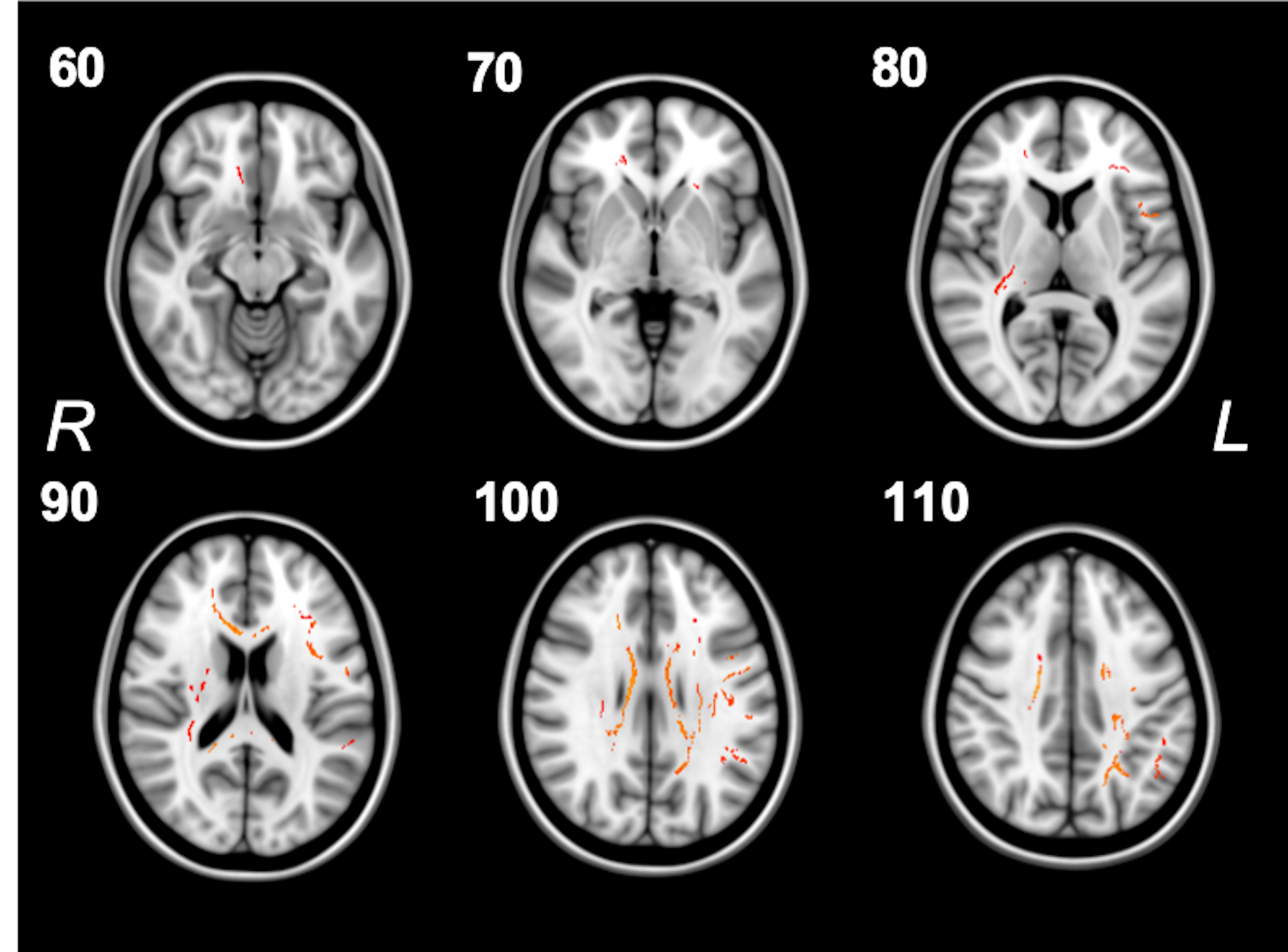

Supplement: Figure S1 [file Image_1.TIFF]
